# Systematic analysis of mushroom body-innervating dopaminergic neuron activity in different physiological states in *Drosophila*

**DOI:** 10.1016/j.bj.2025.100907

**Published:** 2025-08-14

**Authors:** Peng-Shiuan Lee, Meng-Hsuan Chiang, Tony Wu, Chia-Lin Wu

**Affiliations:** a Department of Biomedical Sciences, College of Medicine, Chang Gung University, Taoyuan, Taiwan; b Graduate Institute of Biomedical Sciences, College of Medicine, Chang Gung University, Taoyuan, Taiwan; c Department of Biochemistry, College of Medicine, Chang Gung University, Taoyuan, Taiwan; d Department of Neurology, New Taipei Municipal TuCheng Hospital, Chang Gung Memorial Hospital, New Taipei City, Taiwan; e Brain Research Center, National Tsing Hua University, Hsinchu, Taiwan

**Keywords:** Dopaminergic neuron, Mushroom body, Tyrosine hydroxylase, Thirst, Hunger, Neural activity

## Abstract

**Background:**

Thirst and hunger are fundamental survival drives that modulate various aspects of animal behavior through specific neural circuits. Previous studies have demonstrated that dopaminergic neurons (DANs) innervating the mushroom body (MB) in the *Drosophila* brain play essential roles in innate and learned thirst- and hunger-dependent behaviors, with most experiments focusing on acute water or food deprivation. However, it is unclear whether acute water or food deprivation alters dopamine production and neural activity in MB-innervating DANs.

**Material and methods:**

We genetically expressed green fluorescent protein (GFP) in MB-innervating DANs using broadly and specifically labeled GAL4 lines under satiety, thirst, and hunger states. The brains were immunostained with anti-tyrosine hydroxylase (TH) to assess dopamine biosynthesis. Additionally, the transcriptional reporter of intracellular Ca^2+^ (TRIC) was expressed in these DANs using the same GAL4 lines to monitor neural activity under different internal states. Normalized anti-TH and TRIC signals in specific MB compartments were compared between the satiety and thirst groups and between the satiety and hunger groups using unpaired two-tailed *t*-tests.

**Results:**

Neither TH levels nor neural activity in the 13 subtypes of MB-innervating DANs exhibited significant differences during the satiety, thirst, and hunger conditions.

**Conclusion:**

This study suggests that 16-h water deprivation or 24-h food deprivation does not significantly alter dopamine production and neural activity in MB-innervating DANs. These findings offer insights into the independence of baseline dopaminergic activity from internal states in thirst- or hunger-related behaviors.

## 1 Introduction

Thirst and hunger are fundamental motivational states elicited by deficits in fluid and energy, respectively, which drive animals to make adaptive decisions for survival. These state-dependent behaviors are flexibly regulated by neuromodulators [[1], [2], [3]]. Dopamine has been implicated in modulating thirst- and hunger-dependent behaviors across a wide range of species, from *Drosophila* and zebrafish to mammals [[4], [5], [6], [7], [8]]. However, few studies have focused on how acute thirst or hunger regulates dopamine production and neural activity in the absence of external stimuli [4,5].

*Drosophila* is suitable for creating a model aimed at elucidating the neural mechanisms underlying complex behaviors because of its relatively simple nervous system, availability of abundant genetic tools, and approximately 60% genetic homology with humans [9]. In the *Drosophila* brain, dopaminergic neurons (DANs) that innervate the mushroom body (MB), traditionally regarded as the central hub for olfactory memory, are broadly categorized into protocerebral anterior medial (PAM) and protocerebral posterior lateral (PPL1) neurons based on their axonal projections [10]. Specific subsets of MB-innervating DANs are involved in innate and learned thirst- and hunger-dependent behaviors [[3], [11], [12], [13], [14], [15], [16], [17], [18], [19], [20], [21], [22], [23], [24], [25], [26], [27], [28]]. Because most of these behaviors are related to environmental stimuli, previous studies have primarily analyzed MB-innervating DAN activity in response to external cues such as odors, water, or food [[13], [14], [15],^17^,^18^,^27^,^29^]. In innate behaviors, study has shown that vinegar odor activated PAM-β′2 to reduce aversion to unpleasant CO_2_ odor in hungry flies [14]. In water- or sugar-reward memories, thirsty or hungry flies are trained to associate an odor with a water or sugar reward, and specific MB-innervating DAN activity plays crucial role for reinforcement during association [13,15,18,27]. For water-reward memory, PAM-γ4 is required short-term memory (STM) formation, while PAM-β′1 is essential for long-term memory (LTM) [13,18]. For sugar-reward memory, STM requires PAM-β′2 and PAM-γ4, whereas LTM requires PAM-α1, PAM-β1, PAM-β2, and PAM-γ5 [15,27]. Furthermore, different subsets of MB-innervating DANs have shown distinct response patterns to specific odor cues under different internal states such as hunger [29]. While these findings reveal that thirst or hunger modulates DAN activity under sensory stimuli, it remains unknown whether thirst or hunger is sufficient to affect dopamine release.

In addition, studies have shown that DANs are involved in state-dependent behaviors under experimental paradigms that do not deliberately introduce external stimuli [[19], [20]]. One study identified six MB-innervating DANs associated with food-seeking behavior and investigated whether their activity is directly modulated by hunger. Due to individual differences by GCaMP method, study used yeast odor as an alternative approach and found that only PPL1-γ1pedc and PPL1-α3 showed altered activity in hungry flies [20]. Therefore, it is necessary to clarify how internal states modulate MB-innervating DANs dynamics.

A previous study used the TANGO transgene to examine dopamine release in the MB β and γ lobes, and no significant difference in dopamine release was found between sated and hungry flies [30]. The method lacked systematicity and specificity to resolve dopamine release at the level of defined dopaminergic subpopulations. Herein, we systematically analyzed tyrosine hydroxylase (TH), the rate-limiting enzyme in dopamine biosynthesis and neural activity in 13 subtypes of MB-innervating DANs projecting to 15 distinct MB regions under satiety, thirst, and hunger conditions. We genetically expressed green fluorescent protein (GFP) in MB-innervating DANs using both broadly and specifically labeled GAL4 lines and performed immunostaining against TH in the fly brain in different internal states. TH catalyzes the conversion of tyrosine to l-DOPA, which is subsequently converted into dopamine by DOPA decarboxylase [31]. Although TH indirectly indicates dopamine production, it does not provide information about dopamine release. To overcome this limitation, we assessed neural activity through intracellular calcium, which plays an important role in triggering synaptic vesicle release. We expressed the transcriptional reporter of intracellular Ca^2+^ (TRIC) transgene in DANs using broadly and specifically labeled GAL4 lines. The TRIC uses intracellular Ca^2+^ as an indicator of neural activity [32]. This study provides a subtype-resolved analysis of dopamine production and neural activity in MB-innervating DANs under acute thirst or hunger without sensory stimulation.

## 2 Materials and methods

### 2.1 Fly strains

The flies were raised on standard cornmeal diet at 25°C and 60% relative humidity under a 12 h:12 h light-dark cycle, unless stated otherwise. The experiments were performed using 7-day-old adult female flies. To induce thirst, the flies were deprived of water for 16 h using a 6 cm × 3 cm dry sucrose filter paper in a plastic tube. To induce hunger, flies were deprived of food for 24 h using a 6 cm × 3 cm wet filter paper in a plastic tube. The following fly strains were used in this study: *MB299B*-*GAL4* (Bloomington *Drosophila* Stock Center, BDSC: 68310), *MB063B*-*GAL4* (BDSC: 68248), *MB056B*-*GAL4* (BDSC: 68276), *MB441B*-*GAL4* (BDSC: 68251), *MB312B*-*GAL4* (BDSC: 68314), *MB313C*-*GAL4* (BDSC: 68315), *MB438B*-*GAL4* (BDSC: 68326), *MB296B*-*GAL4* (BDSC: 68308), *MB058B*-*GAL4* (BDSC: 68278), *MB060B*-*GAL4* (BDSC: 68279), *MB304B*-*GAL4* (BDSC: 68367), *R58E02-GAL4* (BDSC: 41347), *MB209B*-*GAL4* (obtained from Suewei Lin's Lab), *TH*-*GAL4* (obtained from Ann-Shyn Chiang's Lab), *VT8167*-*GAL4* (obtained from Ann-Shyn Chiang's Lab), *20XUAS-IVS-mCD8::GFP* (BDSC: 32194), and TRIC (BDSC: 61679).

### 2.2 Immunohistochemistry and brain imaging

The fly brains were dissected in isotonic phosphate-buffered saline (PBS) and fixed in 4% paraformaldehyde (PFA) on ice, enhanced by three rounds of microwave irradiation (2450 MHz; 1100 W) for 1 min each. Subsequently, a second fixation in 4% PFA with 0.25% Triton X-100 was performed under the same conditions. After fixation, the fly brains were transferred to blocking buffer (PBS containing 2% Triton X-100 and 10% normal goat serum) and degassed in a vacuum chamber for four cycles (depressurized to 270 mmHg for 10 min per cycle). Subsequently, the brains were incubated in fresh blocking buffer at room temperature (25°C) for 2 h to ensure permeabilization and blocking. For primary antibody staining, the fly brains were incubated in 1:10 mouse dilution of 4F3 anti-disc large (DLG) monoclonal antibody (AB-528203, Developmental Studies Hybridoma Bank, University of Iowa) or 1:200 dilution of mouse anti-TH monoclonal antibody (22941, ImmunoStar) overnight at room temperature. Following overnight incubation, the fly brains were washed three times with PBS containing 0.1% Triton X-100 (PBS-T) for 20 min each and incubated in 1:200 goat anti-mouse IgG secondary antibody (B-2763, Thermo Fisher Scientific) overnight at room temperature. After three more washes, the fly brains were incubated in 1:500 Alexa Fluor 633 (S21375, Invitrogen) overnight at room temperature. After the final three washes, the fly brains were cleared and mounted with FocusClear (FC-101, CelExplorer) or RapiClear 1.47 (RC147, SUNJin Lab). Imaging was performed using a Zeiss LSM 700 confocal laser scanning microscope equipped with a 40 × water-immersion objective lens (numerical aperture = 1.2, working distance = 220 μm). The pinhole (optical section) was set at 2 μm for images acquired with the 40 × objective lens. All image processing was performed using ZEN software (Zeiss). For the statistical analysis of anti-TH, the anti-TH immunoreactive signals in the GFP-positive regions of the MB lobes were normalized to the anti-TH immunoreactive signals in the ellipsoid body or ventrolateral protocerebrum. For statistical analysis of the TRIC, intracellular calcium levels in the MB lobes were calculated as the ratio of GFP to red fluorescent protein (RFP) signals.

### 2.3 Statistical analysis

Raw data were analyzed using Prism 9 software (version 9.5.0). Data comparing the two groups of internal states were evaluated using unpaired two-tailed *t* tests. Statistical significance was set at *P* < 0.05. The *N* values are shown in the figures. All data are presented as the mean ± standard error of the mean (SEM).

## 3 Results

### 3.1 Tyrosine hydroxylase levels do not change in MB-innervating DANs in thirsty or hungry flies

**Fig. 1:**
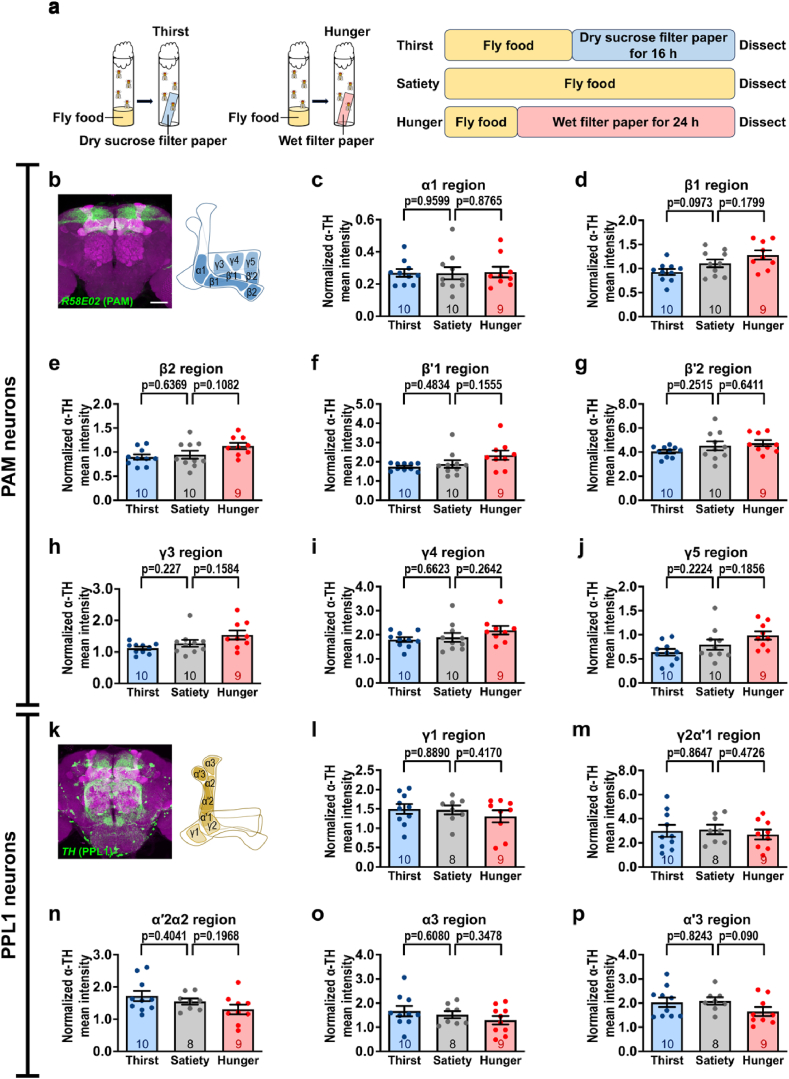
Quantification of TH levels in each MB lobe subdomain by *R58E02*-*GAL4* and *TH*-*GAL4* in thirsty or hungry flies. **(a)** Experimental procedure for generating sated, thirsty, and hungry flies. Flies were initially maintained on standard fly food. For inducing thirst, the flies were provided with a sucrose-soaked filter paper for 16 h in a plastic tube. For inducing hunger, the flies were provided a wet filter paper for 24 h in a plastic tube. **(b)** PAM neurons (green) in the fly brain were labeled using *R58E02*-*GAL4*. The brains were counterstained with anti-DLG antibody (magenta). Scale bar: 50 μm **(c**–**j)** Quantification of anti-TH immunoreactive signals in GFP-positive regions, including α1 **(c)**, β1 **(d)**, β2 **(e)**, β′1 **(f)**, β′2 **(g)**, γ3 **(h)**, γ4 **(i)**, and γ5 **(j)** regions in *R58E02*-*GAL4*/*UAS*-*IVS*-*mCD8*::*GFP* flies under satiety, thirst, and hunger. **(k)** PPL1 neurons (green) in the fly brain were labeled using the *TH*-*GAL4* driver. Brains were counterstained with anti-DLG antibody (magenta). Scale bar: 50 μm **(l**–**p)** Quantification of anti-TH immunoreactive signals in GFP-positive regions, including γ1 **(l)**, γ2α′1 **(m)**, α′2α2 **(n)**, α3 **(o)**, and α′3 **(p)** regions in *TH*-*GAL4*/*UAS*-*IVS*-*mCD8*::*GFP* flies in the satiety, thirst, and hunger conditions. Each number inside the bars indicates the sample size. All the anti-TH immunoreactive signals in GFP-positive MB lobe regions were normalized to the anti-TH immunoreactive signals in the ellipsoid body. Data are presented as mean ± standard error of mean with dots representing individual values. Data were analyzed using unpaired two-tailed *t*-tests.

First, we investigated whether thirst or hunger influenced TH levels in MB-innervating DANs. Previous studies have induced thirst in flies by water deprivation for 16 h and hunger by food deprivation for 16–20 h [13,18,[33], [34], [35]]. In this study, the flies were deprived of water for 16 h and food for 24 h, respectively [Fig. 1a], whereas the sated flies were maintained on a standard diet. These treatments ensured that the flies were in a stable state of thirst or hunger. We first selected the *R58E02*-*GAL4* and *TH*-*GAL4* lines, as these lines are used to broadly label PAM and PPL1 neuron clusters, respectively [[10], [15], [36]]. *R58E02*-*GAL4* labels PAM neurons projecting to the α1, β1, β2, β′1, β′2, γ3, γ4, and γ5 regions of the MB lobes, while *TH*-*GAL4* labels PPL1 neurons innervating the γ1, γ2α′1, α′2α2, α3, and α′3 regions [Fig. 1b and k]. First, we genetically expressed GFP in flies carrying *R58E02*-*GAL4* or *TH*-*GAL4* to visualize the expression of the majority of DANs in each MB lobe compartment. We then immunostained the brains of sated, thirsty, and hungry flies using an anti-TH antibody, and normalized the TH levels in specific MB regions to the TH-positive signal in the ellipsoid body. The ellipsoid body was used as a normalization reference due to its strong TH-positive signal, and its TH levels did not show significant changes across satiety, thirst, and hunger states. Our results showed that TH levels did not significantly change in each MB component labeled with either *R58E02*-*GAL4* or *TH*-*GAL4* in sated, thirsty, or hungry flies [Fig. 1c–j, l-p] [Supplementary Fig. 1].

### 3.2 Tyrosine hydroxylase levels do not change in the MB-innervating PAM and PPL1 neuron subsets in thirsty or hungry flies

**Fig. 2:**
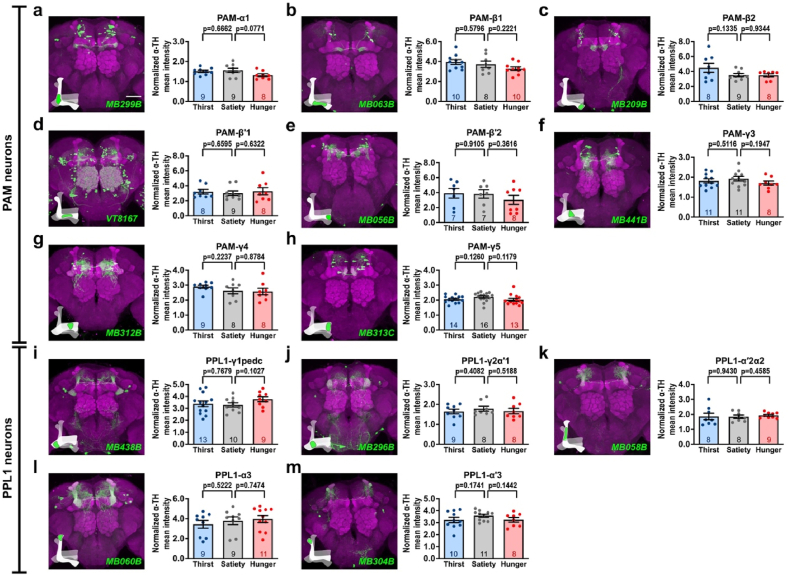
Quantification of TH levels in each MB subdomain by the specific PAM and PPL1 neuron subset GAL4 in thirsty or hungry flies. **(a**–**m)** The brain images in the left panel were counterstained with anti-DLG antibody (magenta). Scale bar: 50 μm. **(a**–**m)** Quantification of anti-TH immunoreactive signals in GFP-positive regions derived using specific GAL4, including PAM-α1 (*MB299B*-*GAL4*) **(a)**, PAM-β1 (*MB063B*-*GAL4*) **(b)**, PAM-β2 (*MB209B*-*GAL4*) **(c)**, PAM-β′1 (*VT8167*-*GAL4*) **(d)**, PAM-β′2 (*MB056B*-*GAL4*) **(e)**, PAM-γ3 (*MB441B*-*GAL4*) **(f)**, PAM-γ4 (*MB312B*-*GAL4*) **(g)**, PAM-γ5 (*MB313C*-*GAL4*) **(h),** PPL1-γ1pedc (*MB438B*-*GAL4*) **(i)**, PPL1-γ2α′1 (*MB296B*-*GAL4*) **(j)**, PPL1-α′2α2 (*MB058B*-*GAL4*) **(k)**, PPL1-α3 (*MB060B*-*GAL4*) **(l)**, and PPL1-α′3 (*MB304B*-*GAL4*) **(m)** during the satiety, thirst, and hunger states. Each number inside the bars indicates the sample size. All the anti-TH immunoreactive signals in GFP-positive MB lobe regions were normalized to the anti-TH immunoreactive signals in the ventrolateral protocerebrum. Data are presented as mean ± standard error of mean with dots representing individual values. Data were analyzed using unpaired two-tailed *t*-tests.

To minimize errors when quantifying TH levels in broadly expressed GAL4 lines, we conducted further experiments using 13 GAL4 lines that specifically labeled subsets of the PAM and PPL1 neurons. The selected GAL4 lines and their corresponding expression patterns were as follows: *MB299B*-*GAL4* labels PAM-α1, *MB063B*-*GAL4* labels PAM-β1, *MB209B*-*GAL4* labels PAM-β2, *VT8167*-*GAL4* labels PAM-β′1, *MB056B*-*GAL4* labels PAM-β′2, *MB441B*-*GAL4* labels PAM-γ3, *MB312B*-*GAL4* labels PAM-γ4, *MB313C*-*GAL4* labels PAM-γ5, *MB438B*-*GAL4* labels PPL1-γ1pedc, *MB296B*-*GAL4* labels PPL1-γ2α′1, *MB058B*-*GAL4* labels PPL1-α′2α2, *MB060B*-*GAL4* labels PPL1-α3, and *MB304B*-*GAL4* labels PPL1-α′3 [10] [Fig. 2]. We genetically expressed GFP in flies carrying each of the above-mentioned GAL4 lines. Thereafter, we immunostained the brains of sated, thirsty, and hungry flies using an anti-TH antibody, and normalized the TH levels in specific MB regions to the TH-positive signal in the ventrolateral protocerebrum. We only analyzed the TH levels in individual subdomains of the MB lobe compartments that were co-localized with GFP signals. Consistent with our initial findings, there was no significant difference in TH levels in each compartment of the individual MB lobe subset in sated, thirsty, or hungry flies [Fig. 2] [Supplementary Fig. 2]. These results suggest that thirst or hunger states does not change TH levels, implying that dopamine biosynthesis is not affected by different internal states [Fig. 2] [Supplementary Fig. 2].

### 3.3 Neural activity does not change in each MB-innervating PAM and PPL1 neuron subset under thirst or hunger states

**Fig. 3:**
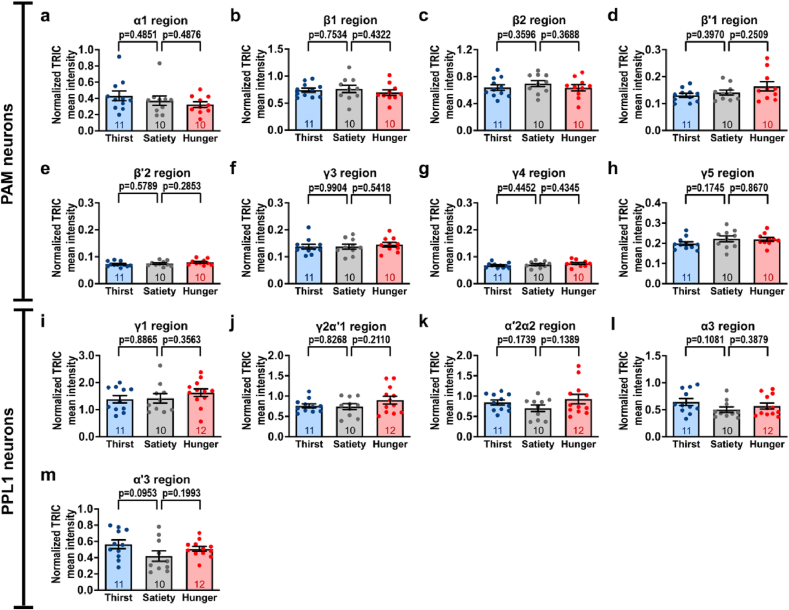
Quantifications of TRIC signals in each MB subdomain by *R58E02*-*GAL4* and *TH*-*GAL4* in thirst or hungry flies. **(a**–**h)** Quantification of TRIC signals (GFP) in RFP-positive regions, including α1 **(a)**, β1 **(b)**, β2 **(c)**, β′1 **(d)**, β′2 **(e)**, γ3 **(f)**, γ4 **(g)**, and γ5 **(h)** regions in +/*10XUAS*-*IVS*-*mCD8*::*RFP*,*13XLexAop2*-*mCD8*::*GFP*;*R58E02*-*GAL4*/*nSyb*-*MKII*::*nlsLexADBDo*; +/*UAS*-*p65AD*::*CaM* flies during the satiety, thirst, and hunger conditions. **(i**–**m)** Quantification of TRIC signals (GFP) in RFP-positive regions, including γ1 **(i)**, γ2α′1 **(j)**, α′2α2 **(k)**, α3 **(l)**, and α′3 **(m)** regions in +/*10XUAS*-*IVS*-*mCD8*::*RFP*,*13XLexAop2*-*mCD8*::*GFP*;+/*nSyb*-*MKII*::*nlsLexADBDo*;*TH*-*GAL4*/*UAS*-*p65AD*::*CaM* flies during satiety, thirst, and hunger. Each number inside the bars indicates the sample size. All GFP signals were normalized to the RFP signals in individual MB lobe compartments. Data are presented as mean ± standard error of mean with dots representing individual values. Data were analyzed using unpaired two-tailed *t*-tests.

Our TH-immunostaining data suggested that the dopamine levels did not change under thirst or hunger states compared with the sated state [Fig. 1, Fig. 2]. Although dopamine production remained unchanged, it was still unclear whether the activity of these DANs was affected by acute thirst or hunger. To evaluate whether thirst or hunger alters neural activity in MB-innervating DANs, we utilized the TRIC transgene, a genetic tool that can monitor long-term changes in neural activity [32]. We genetically expressed TRIC in the *R58E02*-*GAL4* and *TH*-*GAL4* lines, which broadly label PAM and PPL1 neurons, respectively. Thereafter, we dissected the fly brains in satiety, thirst, and hunger states simultaneously and quantified TRIC signals in specific regions of interest by normalizing activity-dependent GFP to endogenously expressed RFP. The results showed that neural activity did not significantly change in each PAM or PPL1 neuron subset labeled by either *R58E02*-*GAL4* or *TH*-*GAL4* under thirst or hunger states [Fig. 3] [Supplementary Fig. 3].

**Fig. 4:**
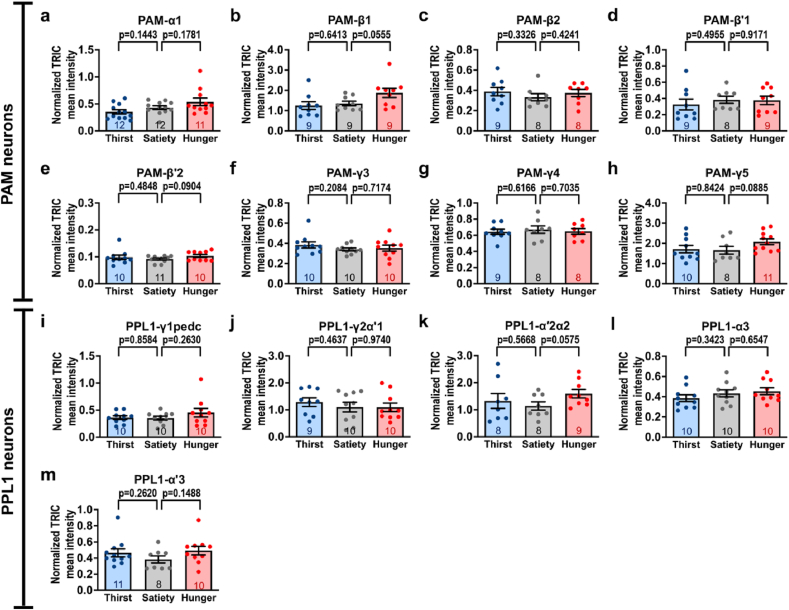
Quantifications of TRIC signals in each MB subdomain by the specific PAM and PPL1 neuron subset GAL4 in thirsty or hungry flies. **(a**–**m)** Quantification of TRIC signals (GFP) in RFP-positive regions which were derived using specific GAL4, including PAM-α1 (*MB299B*-*GAL4*) **(a)**, PAM-β1 (*MB063B*-*GAL4*) **(b)**, PAM-β2 (*MB209B*-*GAL4*) **(c)**, PAM-β′1 (*VT8167*-*GAL4*) **(d)**, PAM-β′2 (*MB056B*-*GAL4*) **(e)**, PAM-γ3 (*MB441B*-*GAL4*) **(f)**, PAM-γ4 (*MB312B*-*GAL4*) **(g)**, PAM-γ5 (*MB313C*-*GAL4*) **(h),** PPL1-γ1pedc (*MB438B*-*GAL4*) **(i)**, PPL1-γ2α′1 (*MB296B*-*GAL4*) **(j)**, PPL1-α′2α2 (*MB058B*-*GAL4*) **(k)**, PPL1-α3 (*MB060B*-*GAL4*) **(l)**, and PPL1-α′3 (*MB304B*-*GAL4*) **(m)** during satiety, thirst, and hunger. Each number inside the bars indicates the sample size. All GFP signals were normalized to the RFP signals in individual MB lobe compartments. Data are presented as mean ± standard error of mean with dots representing individual values. Data were analyzed using unpaired two-tailed *t*-tests.

To minimize errors when quantifying GFP-positive signals in broadly expressed GAL4 lines, we conducted experiments using 13 GAL4 lines that specifically label subsets of PAM and PPL1 neurons and analyzed region-specific neural activity changes under different internal states [Fig. 2]. We genetically expressed TRIC in flies carrying each of the above GAL4 lines. Subsequently, we simultaneously dissected the fly brains under satiety, thirst, and hunger states and quantified TRIC signals in specific regions of interest by normalizing activity-dependent GFP to endogenously expressed RFP. Our results found no significant changes in the TRIC signals in each compartment of the MB lobe subset in sated, thirsty, or hungry flies [Fig. 4] [Supplementary Fig. 4]. These results suggest that the neural activity in each MB-innervating DAN is unchanged under thirst or hunger states.

## 4 Discussion

Water and sugar reward are widely used as reinforcements for olfactory associative memories that require distinct MB-innervating DANs [13,18,[33], [34], [35],^37^,^38^]. This behavioral analysis requires an internal thirst or hunger state to provide the motivation to seek water or food that manifests as different memory expressions [13,18,[33], [34], [35],^37^,^38^]. However, few studies have directly analyzed changes in dopamine levels and neural activity in MB-innervating DANs without external stimulation, to determine whether these DANs are directly affected by thirst or hunger [12,25,30]. In this study, we systematically analyzed the TH levels and neural activity in each DAN neuron cluster innervating the MB lobes. We expressed GFP and TRIC transgenes using the GAL4/UAS binary system with a broadly labeled PAM GAL4 line, broadly labeled PPL1 GAL4 line, and 13 specific GAL4 lines labeled with individual DAN subsets for immunostaining, quantification, and analysis of regions of interest in the MB lobes. The results showed no significant differences in TH levels and DAN activity in the satiety, thirst, and hunger states [Fig. 1, Fig. 2, Fig. 3, Fig. 4] and [Supplementary Figs. 1–4], suggesting that dopamine levels and neural activity in MB-innervating DANs are not influenced by thirst or hunger without stimuli from the outside environment.

The activity of MB-innervating DANs changes dynamically in response to different physiological states and environmental inputs [[13], [14], [15],^17^,^18^,^27^,^29^]. Our results did not show a significant change in TH levels in MB-innervating DANs. This may be because the MB-innervating DANs synthesize sufficient dopamine in advance, allowing them to dynamically adjust their activity in response to various environmental stimuli. This regulation may arise from different upstream inputs to MB-innervating DANs [17]. Appetite memory formation is regulated by the inhibition of PAM-γ3 by the satiety-signaling neuropeptide allatostatin (AstA) when hungry flies consume food [17]. Thus, our experiment did not find a significant change in the TH level of PAM-γ3 [Fig. 2f].

A prior study reported a decrease in PPL1-γ1pedc neural activity after 24 h of hunger compared with satiety using the calcium-dependent nuclear import of LexA (CaLexA) transgene via *MB320C*-*GAL4* [25]. When we expressed TRIC in PPL1-γ1pedc using *MB438B*-*GAL4*, we observed no significant difference in neural activity between satiety and 24 h of hunger [Fig. 4i] and [Supplementary Fig. 4i]. The discrepancies in the previous and current studies’ findings may be attributed to variations in the genetic tools, sample sizes, and statistical methods. Another study has indicated that leucokinin (LK) neurons exhibit long-term activity during thirst or hunger. LK inhibits PAM-β′2a and PPL1-γ2α′1 to promote water-reward memory expression and activates PAM-β′2mp to promote sugar-reward memory expression [22]. In this study, we detected TH levels without external stimuli [Fig. 2e and j] and [Supplementary Fig. 2e and 2j], implying that LK neurons influence dopamine transmission by releasing LK peptides without affecting TH levels in the DANs during thirst or hunger. However, we did not observe significant changes in neural activity in PPL1-γ2α′1 during thirst [Fig. 4j] and [Supplementary Fig. 4j] or PAM-β′2 during hunger [Fig. 4e] and [Supplementary Fig. 4e]. Since TRIC relies on the gradual accumulation of GFP at the cell membrane, it captures sustained neural activity over a long period rather than rapid changes. Over longer timescales, DANs may receive LK signals as well as other modulatory inputs. These additional factors could have masked DAN activity in the TRIC experiment, leading to the lack of observed significant changes.

The early and late phases of acute water or food deprivation, as well as the contrast between acute and chronic deprivation, affect behavior and neural regulation through distinct mechanisms [22,39,40]. Acute deprivation refers to a short-term lack of hydration or nutrients, whereas chronic deprivation involves prolonged but partial insufficiency. In flies, 6 h mild and 12 h extreme water deprivation resulted in different levels of thirst-responsive genes in brains [39]. For hunger, LK neurons were activated only after 20 h food deprivation, not after 8 h [22]. While few studies in flies have directly compared acute and chronic deprivation, study in rats showed that 48 h acute food deprivation and chronic restriction to 70% of normal food intake resulted in different neuropeptide Y expression in the hypothalamus [40]. Ample studies analyzing thirst- or hunger-dependent behaviors in *Drosophila* typically use 16–24 h water or food deprivation [13,18,[33], [34], [35]]. Based on our prior work, we found that deprivation within this range is sufficient to induce both behavioral and physiological changes [1,18]. Flies show increased hot avoidance after 24 h food deprivation [1], or form water-reward memory after 16 h water deprivation [18].

The limitation of this study is the use of TH levels as a proxy for dopamine production, without direct assessment of dopamine release due to the lack of a reliable anti-dopamine antibody. TH is a rate-limiting enzyme in dopamine synthesis. We anticipated that an increase in TH levels within specific DANs can lead to an increase in dopamine levels. As an alternative approach, GRAB dopamine sensor has been used to directly measure dopamine release levels [41]. GRAB dopamine sensor can be expressed in DANs or the MB, and upon dopamine binding, the sensor emits an increased green fluorescent signal. Study has used GRAB dopamine sensor to test spontaneous dopamine input to MB γ lobe [24]. Imaging mass spectrometry can also spatially detect dopamine levels in *Drosophila* brain [42]. Nevertheless, we cannot rule out the possibility that the activity of dopaminergic circuits may be regulated via alternative mechanisms involving dopamine metabolism and reuptake under different physiological states. For instance, dopamine levels can be modulated through the phosphorylation-mediated regulation of TH activity [43] or alterations in dopamine reuptake receptor expression [26]. A study has shown that hunger upregulates Dop2R autoreceptor expression on PPL1 axons, thereby suppressing PPL1 output and facilitating appetitive memory expression [26]. Additionally, internal states have been shown to influence the synaptic architecture [20]. A study found that hunger increases the density or size of the active zone in PPL1-γ2α′1 and PPL1-α'2α2, without altering odor-evoked calcium transients, suggesting that hunger enhances dopamine secretion levels without altering calcium signaling dynamics [20].

TRIC is used to analyze long-term neural activity, in contrast to GCaMP, which detects rapid calcium transients. Given that physiological states such as thirst or hunger change gradually over time, TRIC is more suitable for capturing sustained neural responses [32]. However, we cannot fully exclude the possibility of brief activity changes that may have gone undetected. An alternative method to capture short-term activity is GCaMP. A previous study used the GCaMP transgene to examine the activity of specific MB-innervating DANs in hungry flies, and reported variability in baseline activity across individual flies [20]. Future studies investigating spontaneous activity change would benefit from normalizing GCaMP signals with an internal control.

Despite the limitations of our experimental methods, we found no significant differences in TH levels and neural activity in MB-innervating DANs under thirst or hunger states, suggesting that acute water or food deprivation does not alter dopamine production and neural activity in MB-innervating DANs. Our results provide a robust framework for investigating how external stimuli influence the activity of these DANs in different physiological states.

## Author contributions

P. –S. L., M.-H. C., and C.-L. W. designed the study. P. –S. L. and M.-H. C. conducted the experiments. P. –S. L., M.-H. C., T. W., and C.-L. W. analyzed the data. P. –S. L., M.-H. C., and C.-L. W. wrote the manuscript.

## Funding sources

This work was supported by grants from 10.13039/100020595National Science and Technology Council, Taiwan (112-2311-B-182-002-MY3, 113-2622-B-182-001, and 114-2311-B-182-002-MY3) to C.-L.W., and grants from 10.13039/100012553Chang Gung Memorial Hospital, Taiwan (CMRPD1M0761-3 and BMRPC75) to C.-L.W.

## Declaration of competing interest

The authors declare no conflicts of interest.
