# Supplementary material for: Systematic analysis of mushroom body-innervating dopaminergic neuron activity in different physiological states in Drosophila
Source: Biomed J. 2025 Aug 14;49(3):100907. doi: 10.1016/j.bj.2025.100907 (PMC13226800; doi:10.1016/j.bj.2025.100907)
Supplement: Multimedia component 1 [file mmc1.docx]

**Supplementary Figures**

**
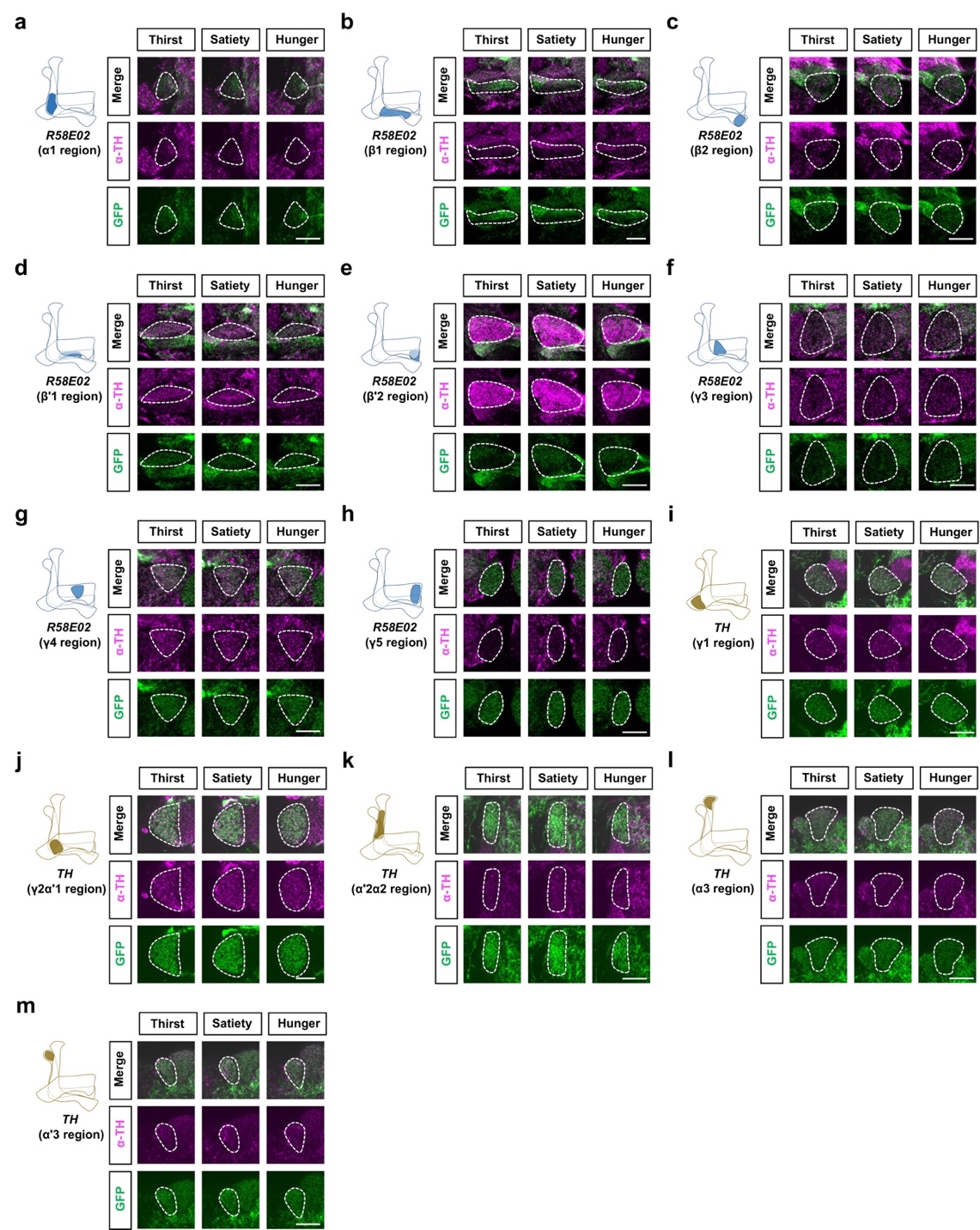
**

**Supplementary Fig. 1. Anti-TH immunoreactive signals in *R58E02*-*GAL4* and *TH*-*GAL4* under different physiological states. (a-h)** Representative images of anti-TH immunoreactive signals in GFP-positive regions of the MB lobes, including α1 **(a)**, β1 **(b)**, β2 **(c)**, β'1 **(d)**, β'2 **(e)**, γ3 **(f)**, γ4 **(g)**, and γ5 **(h)** regions in *R58E02*-*GAL4*/*UAS*-*IVS*-*mCD8*::*GFP* flies during satiety, thirsty, and hunger. **(i-m)** Representative images of anti-TH immunoreactive signals in GFP-positive regions of the MB lobes, including γ1 **(i)**, γ2α'1 **(j)**, α'2α2 **(k)**, α3 **(l)**, and α'3 **(m)** regions in *TH*-*GAL4*/*UAS*-*IVS*-*mCD8*::*GFP* flies during satiety, thirsty, and hunger. Scale bar: 20 μm.


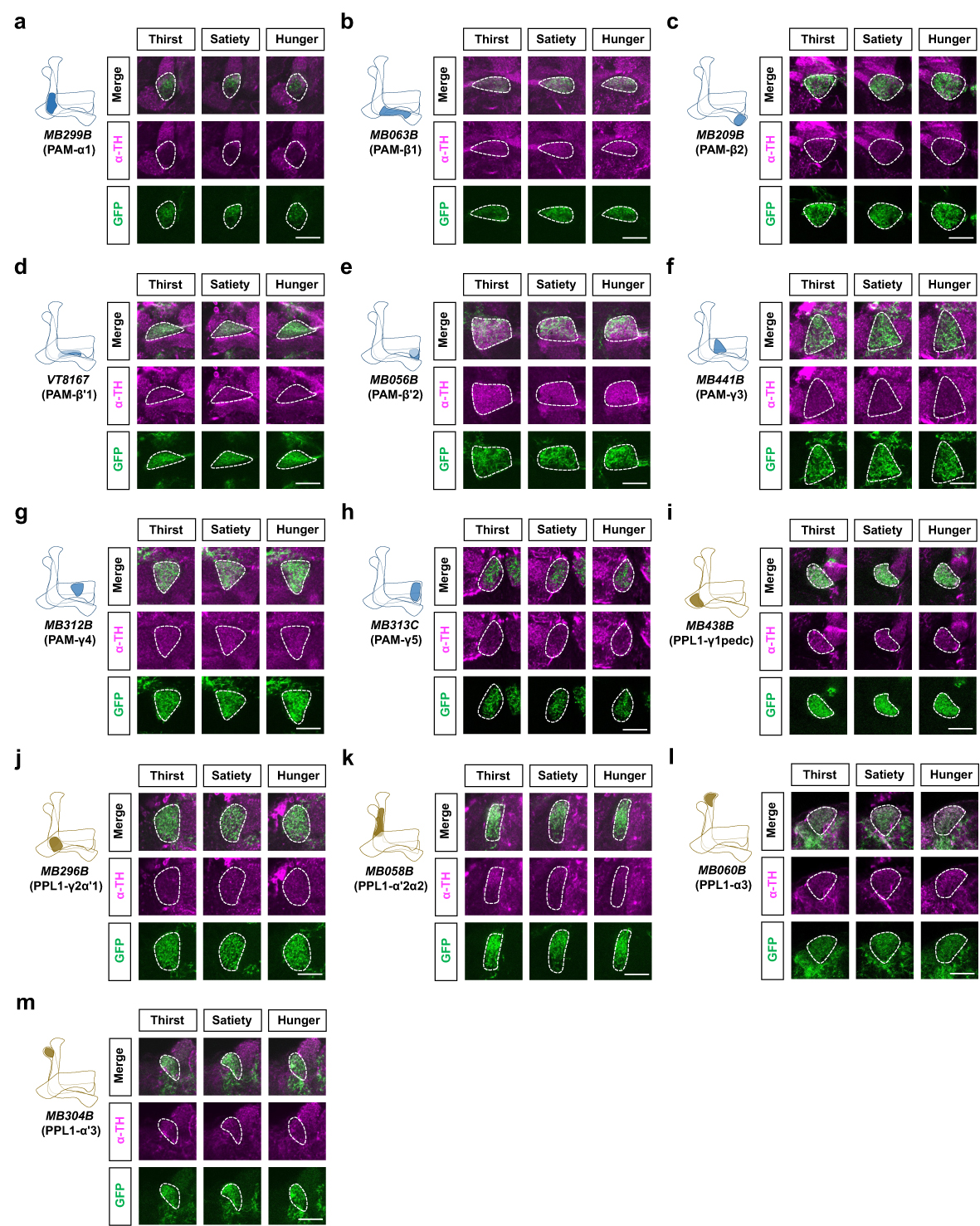


**Supplementary Fig. 2. Anti-TH immunoreactive signals in each PAM and PPL1 neuron subset under different physiological states. (a-m)** Representative images of anti-TH immunoreactive signals in the GFP-positive regions of MB lobes derived using specific GAL4, including PAM-α1 (*MB299B*-*GAL4*) **(a)**, PAM-β1 (*MB063B*-*GAL4*) **(b)**, PAM-β2 (*MB209B*-*GAL4*) **(c)**, PAM-β'1 (*VT8167*-*GAL4*) **(d)**, PAM-β'2 (*MB056B*-*GAL4*) **(e)**, PAM-γ3 (*MB441B*-*GAL4*) **(f)**, PAM-γ4 (*MB312B*-*GAL4*) **(g)**, PAM-γ5 (*MB313C*-*GAL4*) **(h)**, PPL1-γ1pedc (*MB438B*-*GAL4*) **(i)**, PPL1-γ2α'1 (*MB296B*-*GAL4*) **(j)**, PPL1-α'2α2 (*MB058B*-*GAL4*) **(k)**, PPL1-α3 (*MB060B*-*GAL4*) **(l)**, and PPL1-α'3 (*MB304B*-*GAL4*) **(m)** under satiety, thirst, and hunger conditions. Scale bar: 20 μm.

**
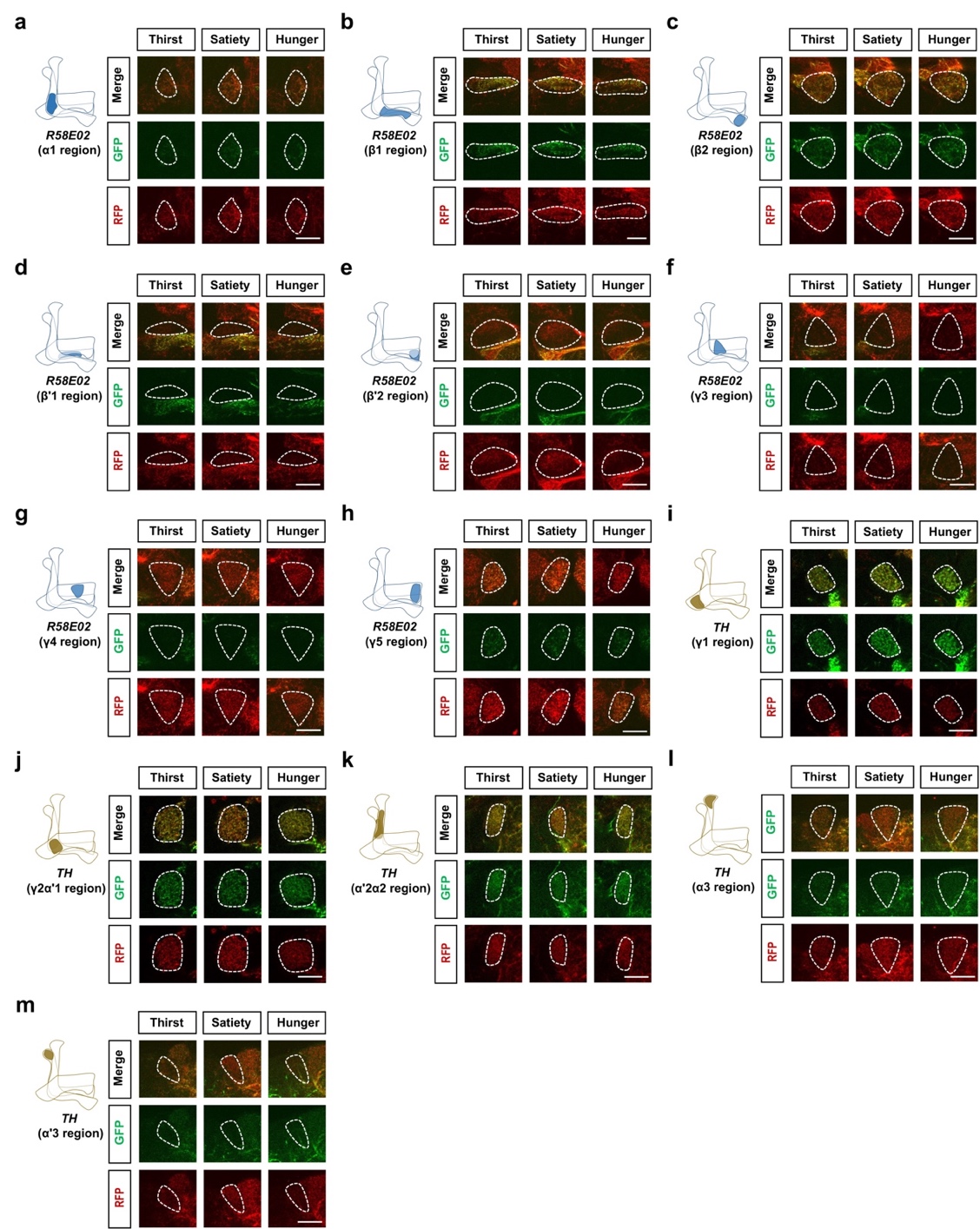
**

**Supplementary Fig. 3. TRIC signals in *R58E02*-*GAL4* and *TH-GAL4* positive regions of the MB lobes under different physiological states. (a-h)** Representative images of TRIC signals (GFP) in the RFP-positive regions in individual MB lobe compartments, including α1 **(a)**, β1 **(b)**, β2 **(c)**, β'1 **(d)**, β'2 **(e)**, γ3 **(f)**, γ4 **(g)**, and γ5 **(h)** regions in +/*10XUAS*-*IVS*-*mCD8*::*RFP*,*13XLexAop2*-*mCD8*::*GFP*;*R58E02*-*GAL4*/*nSyb*-*MKII*::*nlsLexADBDo*; +/*UAS*-*p65AD*::*CaM* flies in the satiety, thirst, and hunger states. **(i-m)** Representative images of TRIC signals in the RFP-positive regions in individual MB lobe compartments, including γ1 **(i)**, γ2α'1 **(j)**, α'2α2 **(k)**, α3 **(l)**, and α'3 **(m)** regions in +/*10XUAS*-*IVS*-*mCD8*::*RFP*,*13XLexAop2*-*mCD8*::*GFP*;*TH*-*GAL4*/*nSyb*-*MKII*::*nlsLexADBDo*; +/*UAS*-*p65AD*::*CaM* flies in the satiety, thirst, and hunger states. Scale bar: 20 μm.

**
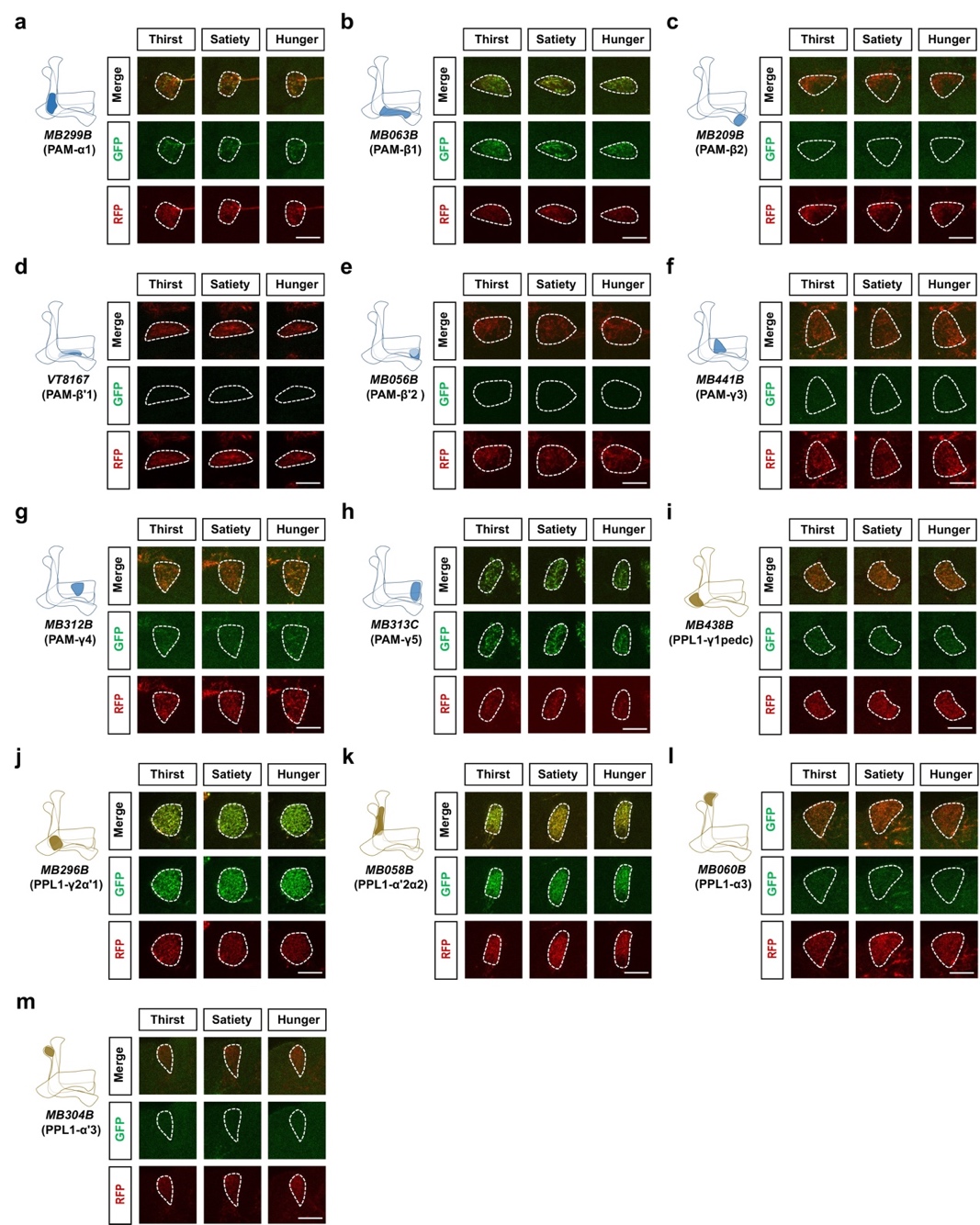
**

**Supplementary Fig. 4. TRIC signals in each PAM and PPL1 neuron subset under different physiological states. (a-m)** Representative images of TRIC signals (GFP) in the RFP-positive regions derived using PAM- or PPL1-specific GAL4, including PAM-α1 (*MB299B*-*GAL4*) **(a)**, PAM-β1 (*MB063B*-*GAL4*) **(b)**, PAM-β2 (*MB209B*-*GAL4*) **(c)**, PAM-β'1 (*VT8167*-*GAL4*) **(d)**, PAM-β'2 (*MB056B*-*GAL4*) **(e)**, PAM-γ3 (*MB441B*-*GAL4*) **(f)**, PAM-γ4 (*MB312B*-*GAL4*) **(g)**, PAM-γ5 (*MB313C*-*GAL4*) **(h)**, PPL1-γ1pedc (*MB438B*-*GAL4*) **(i)**, PPL1-γ2α'1 (*MB296B*-*GAL4*) **(j)**, PPL1-α'2α2 (*MB058B*-*GAL4*) **(k)**, PPL1-α3 (*MB060B*-*GAL4*) **(l)**, and PPL1-α'3 (*MB304B*-*GAL4*) **(m)** in the satiety, thirst, and hunger states. Scale bar: 20 μm.
